# Transgluteal MRI in-bore prostate biopsy in routine clinical practice: a contemporary-cohort-study from a german tertiary center

**DOI:** 10.1007/s11255-025-04983-4

**Published:** 2026-01-02

**Authors:** Thomas Vogl, Maximilian Holzer, Scherwin Mahmoudi, Leon Grünewald, Vitali Koch, Jennifer Gotta, Philipp Reschke, Mike Wenzel, Benedikt Hoeh, Philipp Mandel, Felix Chun, Jens Köllermann, Boris Bodelle, Simon Martin, Jan-Erik Scholtz, Renate Hammerstingl, Tatjana Gruber-Rouh, Katrin Eichler, Peter Wild, Simon Bernatz

**Affiliations:** 1 Goethe University Frankfurt, University Hospital, Clinic for Radiology and Nuclear Medicine, Frankfurt Am Main, Germany; 2 Department of Urology, Goethe University Frankfurt, University Hospital, Frankfurt Am Main, Germany; 3 Department of Urology, University Hospital Carl Gustav Carus, Technische Universität Dresden, Dresden, Germany; 4 Goethe University Frankfurt, University Hospital, Dr. Senckenberg Institute for Pathology, Frankfurt Am Main, Germany; 5 Department of Diagnostic and Interventional Radiology, Klinikum Mutterhaus Der Borromäerinnen, Trier, Germany; 6 Frankfurt Institute for Advanced Studies (FIAS), Frankfurt Am Main, Germany

**Keywords:** Prostate cancer, Magnetic resonance imaging, Image-guided biopsy, PI-RADS, Diagnostic accuracy

## Abstract

**Objectives:**

To assess the diagnostic performance, predictors, and clinical utility of MRI-guided prostate biopsy over an 18 year period in a real-world tertiary care setting.

**Materials and methods:**

We retrospectively analyzed patients who underwent MRI-guided prostate biopsy between 2006 and 2024 at a German tertiary care center. Clinical data, PI-RADS, PSA levels, prostate volume, and biopsy outcomes were evaluated. Logistic regression models assessed associations between clinical variables and cancer detection, clinical significance (ISUP ≥ 3), and upgrading after radical prostatectomy (RPE).

**Results:**

Among 496 patients (mean age 66 ± 8 years; PSA, median (IQR): 7.2 ng/ml (5–10)), cancer was detected in 33% (162/496), with 29% (47/162) classified as clinically significant. In our biopsy cohort (PI-RADS 3: 12.7%, PI-RADS 4: 43.0%, PI-RADS 5: 5.6%), clinically significant prostate cancer (ISUP ≥ 3) was detected in 3.2% of PI-RADS 3, 12.2% of PI-RADS 4, and 28.6% of PI-RADS 5 lesions. PI-RADS 5 lesions were strongly associated with cancer detection (OR 3.6, 95% CI 1.35–10.13) and significant disease (OR 7.1, 95% CI 1.38–55.15), independent of age, prostate volume, and biopsy extent. While these covariates predicted overall biopsy positivity (*p* < 0.02), they were not associated with significant cancer (*p* > 0.05) or upgrading at RPE (*p* > 0.05). Among 29 RPE patients, only one was upgraded. Post-biopsy CT was performed in all patients: MRI-guided biopsy was associated with a low complication rate: minor localized bleeding occurred in 4.4% of cases, and no major adverse events were observed.

**Conclusions:**

MRI-guided prostate biopsy showed high diagnostic accuracy and safety in routine care. PI-RADS was the key predictor of clinically significant cancer. The low rate of upgrading after RPE supports its reliability in guiding patient management and avoiding overtreatment.

**Clinical relevance statement:**

MRI-guided prostate biopsy enables accurate detection of clinically significant prostate cancer with low risk of understaging, supporting its routine use in personalized urologic oncology.

## Introduction

Prostate cancer is the second most frequently diagnosed malignancy among men [1]. Identification of clinically significant disease is essential to optimize patient management and reduce unnecessary morbidity from overdiagnosis and overtreatment [2].

Magnetic Resonance Imaging (MRI) has become an integral part of prostate cancer diagnostics and is now established before biopsy in patients with intermediate risk for cancer, according to the latest European Association of Urology (EAU) guidelines [2, 3]. Multiparametric MRI allows for the detection and localization of lesions with a higher likelihood of clinical relevance, facilitating targeted sampling while reducing the detection of indolent cancers [4]. Image-guided biopsy techniques, particularly MRI-targeted biopsies, have shown superior diagnostic performance in detecting clinically significant prostate cancer compared to systematic biopsy alone [5–8]. Clinically significant prostate cancer (csPCa) is commonly defined as ISUP grade group ≥ 2 (Gleason 3 + 4), reflecting tumors with potential clinical relevance. In this study, we focused on ISUP ≥ 3 (Gleason ≥ 4 + 3) to identify higher-risk individuals with unfavorable intermediate risk or worse, who are more likely to experience disease progression and require definitive therapy rather than active surveillance [9–12]. This approach prioritizes detection of tumors with the greatest clinical impact while reducing the likelihood of overdiagnosis and overtreatment associated with lower-grade cancers, particularly in biopsy-based studies.

Multiparametric MRI enables the detection and localisation of lesions with a higher probability of clinical significance, thereby supporting targeted biopsy while limiting the identification of indolent prostate cancers [13, 14]. Most evidence to date stems from clinical trials or controlled settings that may not reflect the diversity of patients, imaging quality, and procedural expertise encountered in standard care [15]. Furthermore, the extent to which MRI-targeted biopsies prevent understaging, particularly upgrading at radical prostatectomy, remains insufficiently investigated in real-world settings, where clinical decision-making regarding active surveillance, focal therapy, or radical treatment critically depends on accurate biopsy results [16].

MRI-guided prostate biopsy techniques have transformed prostate cancer diagnosis by significantly improving the detection of clinically significant disease compared with conventional ultrasound-guided biopsy. The three principal MRI-targeted approaches: cognitive (visual registration), MRI/ultrasound (US) fusion-guided, and MRI in-bore, differ in precision, operator dependency, and resource requirements. Cognitive biopsy relies on the operator’s mental mapping of MRI-identified lesions onto real-time ultrasound, offering cost-effectiveness and wide accessibility but with variable accuracy and reproducibility [17–21]. MRI/US fusion-guided biopsy employs specialized software to overlay MRI data onto real-time ultrasound, enabling more consistent targeting, lesion tracking, and documentation while reducing operator dependency [19, 21–23]. MRI in-bore biopsy, performed entirely under MRI guidance, provides real-time visualization of both the lesion and biopsy needle, allowing for precise sampling of small or challenging lesions with fewer cores, albeit with greater cost and limited availability [18, 20, 24–26]. Overall, while detection rates for clinically significant prostate cancer are broadly similar across approaches: approximately 37% for cognitive, 39% for fusion-guided, and 47% for in-bore biopsies, differences in resource needs, operator experience, and workflow complexity guide method selection in clinical practice [20, 22, 27, 28]. Within this context, the present study focuses exclusively on MRI in-bore–guided prostate biopsy as the MRI-targeted technique used at our institution. Emerging evidence supports the continued role of all three methods in precision prostate cancer diagnostics, with ongoing comparative studies aimed at defining their optimal use in varied clinical and economic settings [23, 25].

Given the increasing role of MRI in patient counselling and treatment planning, there is an urgent need for real-world data to assess MRI-guided prostate biopsy diagnostic performance and safety profile in routine clinical practice [29, 30]. This is especially relevant as healthcare systems increasingly focus on value-based care and shared decision-making that respects patient quality of life [31].

The aim of this study was to evaluate the diagnostic performance, predictors, and clinical implications of transgluteal MRI in-bore prostate biopsy in a large, real-world cohort over an 18-year period at a tertiary care center. By analyzing cancer detection, the incidence of clinically significant disease, and concordance with prostatectomy specimens, we provide robust evidence on the reliability and clinical value of MRI-guided biopsy in routine practice.

## Methods

This retrospective, single-center cohort study was approved by the institutional review board of Goethe University Frankfurt (approval number 2021–235). The requirement for written informed consent was waived due to the retrospective design. The study complies with the Declaration of Helsinki and relevant national and institutional guidelines for research involving human participants.

### Study design and patient selection

**Fig. 1:**
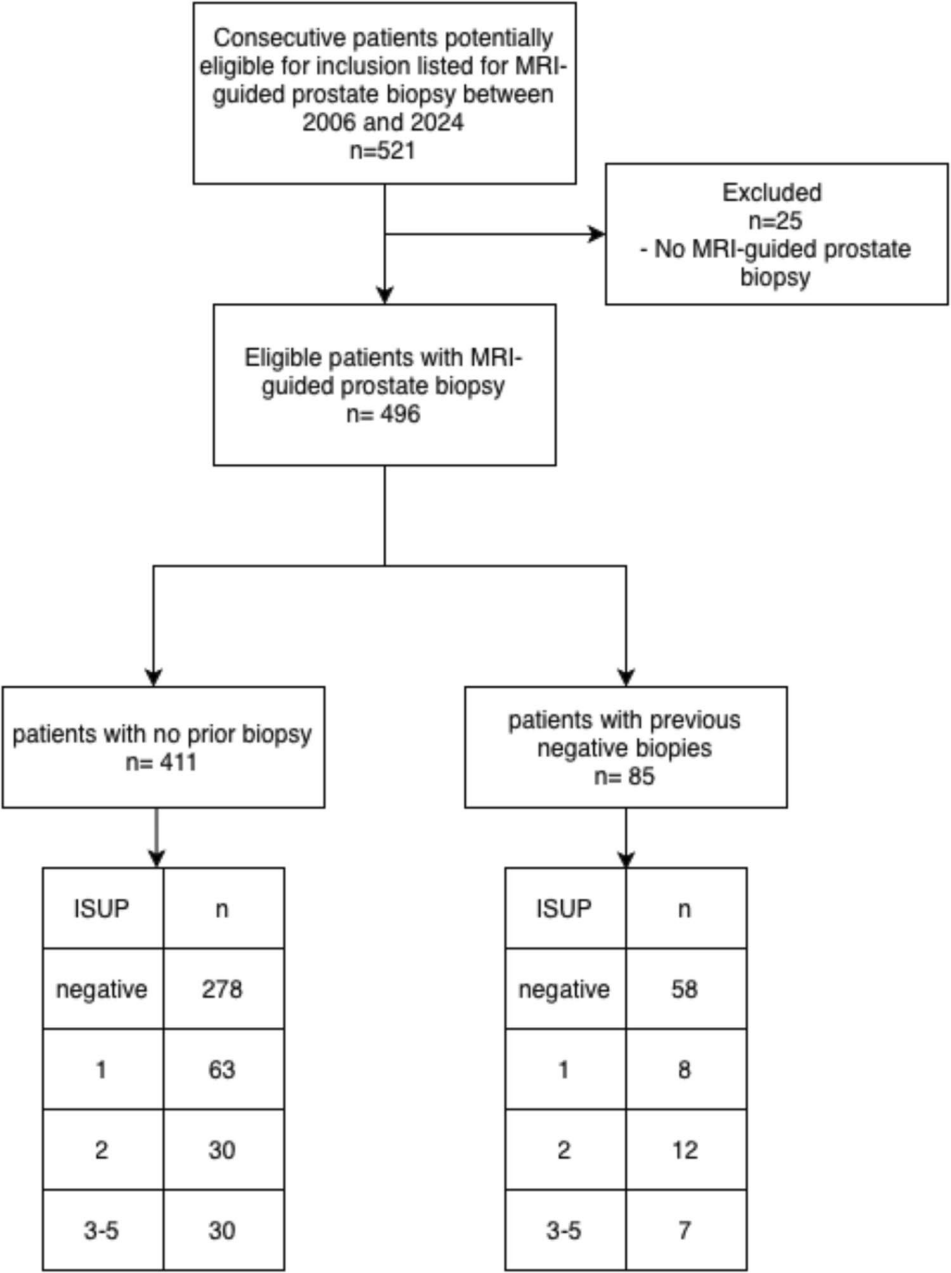
Flowchart of patient inclusion

We retrospectively identified all patients who underwent MRI-guided prostate biopsy between January 2006 and August 2024 at the tertiary academic institution of the university hospital in Frankfurt am Main. Patients were retrieved via institutional radiology databases using procedural codes. Inclusion criteria were (i) availability of MRI-guided prostate biopsy imaging or reports, and (ii) histopathological evaluation. Exclusion criteria were (i) coding errors of patients not having received an MRI-guided biopsy. All included patients were enrolled consecutively without randomization (Fig. 1).

### Biopsy procedure

**Fig. 2:**
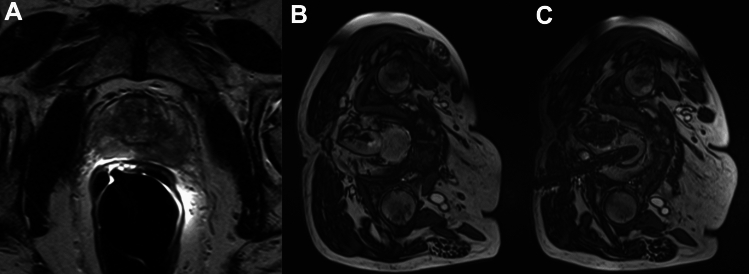
Case Presentation (72 year-old patient) In the current multiparametric MRI of the prostate, the axial T2-weighted sequence **a** demonstrates a left-sided, confluent, ill-defined, hypointense lesion located in the peripheral zone at the level of the prostatic base, suspicious for prostate cancer. For targeted detection and biopsy planning, image **b** shows the planning scan used for biopsy. During tissue sampling, the MR guidance image **c** confirms the correct positioning of the biopsy needle within the left-sided index lesion

After positioning the patient in the lateral decubitus position within the MRI scanner, both superficial and deep anesthesia were administered using 20 ml of Scandicain per side by a specialized radiologist (TV) with over 10 years of experience in interventional radiology. Under MRI guidance using T2-weighted sequences (needle in scan), one needle (inner lumen 4 mm, 18 g needle somatex) was inserted transgluteally through the gluteus maximus muscle into the peripheral zone of the prostate. Following precise positioning of the needle within the suspicious lesion, multiple biopsies were obtained using a biopsy punch (maximum core length: 20 mm). The number of biopsy cores taken per patient was recorded (median 6, IQR 4–12). The mean biopsy time per patient was approximately 25 min (± 5 min). A control CT scan was subsequently performed to exclude post-procedural complications [32] (Fig. 2).

### MRI acquisition and evaluation

Multiparametric prostate MRI was performed in clinical routine using either a 1.5 T Siemens Ära or Avanto scanner with standard body coils. Available MRI scans followed the European Society of Urogenital Radiology (ESUR) guidelines, including T1-weighted (T1w), T2-weighted (T2w), diffusion-weighted imaging (DWI), apparent diffusion coefficient (ADC), and dynamic contrast-enhanced (DCE) sequences. Lesions identified from 2012 onwards were scored using PI-RADS versions 1.0, 2.0, or 2.1 by board-certified radiologists. In clinical practice, the most current version of PI-RADS available at the time of evaluation was consistently applied: v1.0 (during 2012–2014) [33], v2.0 (2015–2018) [34] and v2.1 (from 2019) [29]. Lesions with PI-RADS ≥ 3 were considered potential targets for biopsy.

### Histopathological reference standard

All biopsy specimens were reviewed by board-certified genitourinary pathologists using the ISUP grading system. Clinically significant prostate cancer (csPCa) was defined as ISUP grade ≥ 3. In a subset of patients who underwent in-center radical prostatectomy, surgical pathology served as the reference standard to assess concordance and upgrading.

### Statistical analysis

Continuous variables were summarized as means ± standard deviation (SD); categorical variables were presented as counts and percentages. Logistic regression models were used to assess associations between clinical/imaging variables and the following outcomes: (i) positive biopsy, (ii) csPCa, and (iii) upgrading at radical prostatectomy. Statistical analyses were performed using R version 4.2.2 (R Foundation for Statistical Computing). A two-sided p-value < 0.05 was considered statistically significant.

## Results

### Patient characteristics and biopsy outcomes

A total of 496 patients who underwent MRI-guided prostate biopsy between 2006 and 2024 were included. The mean age was 66 years (IQR 61–71), with a mean prostate volume of 51.1 ml (SD 25.9) and a mean prebiopsy PSA of 7.2 (IQR 5–10). On average, patients had one prior biopsy (mean 1.0, SD 0.85). Cancer was detected in 162 of 496 patients (33%). Of these, 47 cases (29%)were classified as clinically significant prostate cancer (csPCa), defined as ISUP grade ≥ 3. Clinical characteristics are summarized in Table 1. The PI-RADS distribution within the biopsy cohort was as follows: 192 lesions were unclassified (38.7%), 63 were rated PI-RADS 3 (12.7%), 213 PI-RADS 4 (43.0%), and 28 PI-RADS 5 (5.6%).

**Table 1:** Clinical and epidemiological characteristics.

	Overall
n	496
Age at biopsy(median (IQR, range)	66 (61–71, 40–87)
Prostatevolume, ml (mean (SD))	51.09 (25.88)
PSA, total, prebiopsy(median(IQR, range))	7.2 (5–10, 0.35–88.7)
Number of prebiopies (mean (SD))	1.02 (0.85)
positive biopsy (%)	162 (32.7)
significant cancer (%)	47 (29.4)
PI-RADS (%)	
3	63 (20.7)
4	213 (70.1)
5	28 ( 9.2)
ISUP, biopsy (%)	
1	71 (44.4)
2	42 (26.2)
3	16 (10.0)
4	14 ( 8.8)
5	17 (10.6)
ISUP, radical prostatectomy (%)	
1	3 (10.3)
2	15 (51.7)
3	3 (10.3)
4	2 ( 6.9)
5	6 (20.7)
any upgrading (%)	17 (63.0)

Differences in patient count reflect missing values. *ISUP* international society of urological pathology, *PIRADS* prostate imaging reporting and data system

**Fig. 3:**
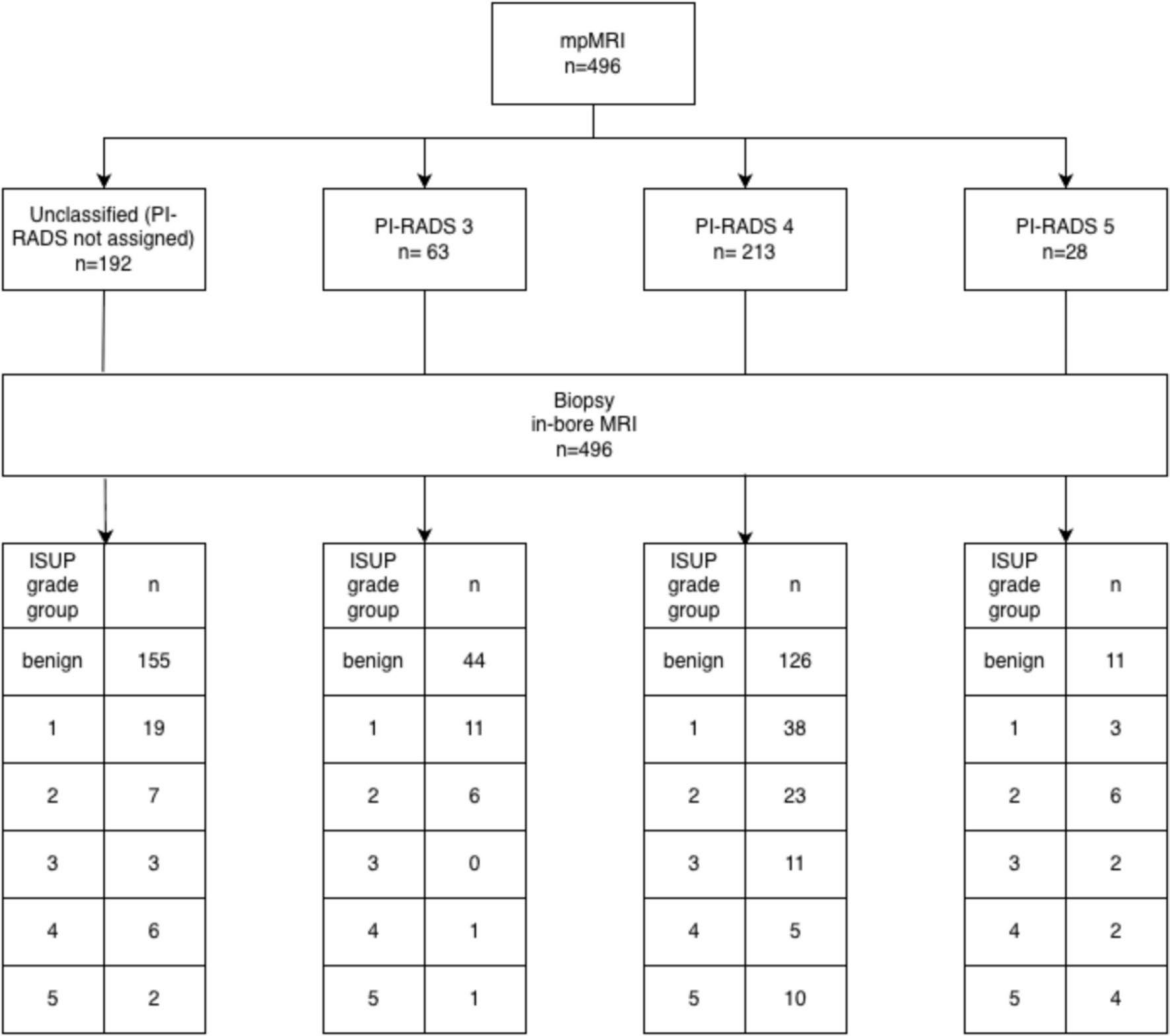
The figure presents a flowchart of all 496 patients who underwent mpMRI, stratified into PI-RADS categories (unclassified, PI-RADS 3, 4, and 5). For each subgroup, the results of in-bore MRI–guided biopsy are summarized, showing the distribution of ISUP grade groups (benign to ISUP 5) with corresponding case numbers. The diagram illustrates how tumor grade varies according to initial PI-RADS assessment

Across all PI-RADS categories, the corresponding ISUP grade distribution on targeted biopsy is summarized in Fig. 3. Using ISUP grade ≥ 3 as the definition for clinically significant prostate cancer (csPCa), the overall detection rates increased with higher PI-RADS categories. In the PI-RADS 3 group, csPCa was identified in 2 of 63 lesions (3.2%). For PI-RADS 4 lesions, 26 of 213 (12.2%) were classified as csPCa. The highest detection rate was observed in the PI-RADS 5 category, in which 8 of 28 lesions (28.6%) were found to harbor csPCa.

### Predictors of positive biopsy

**Fig. 4:**
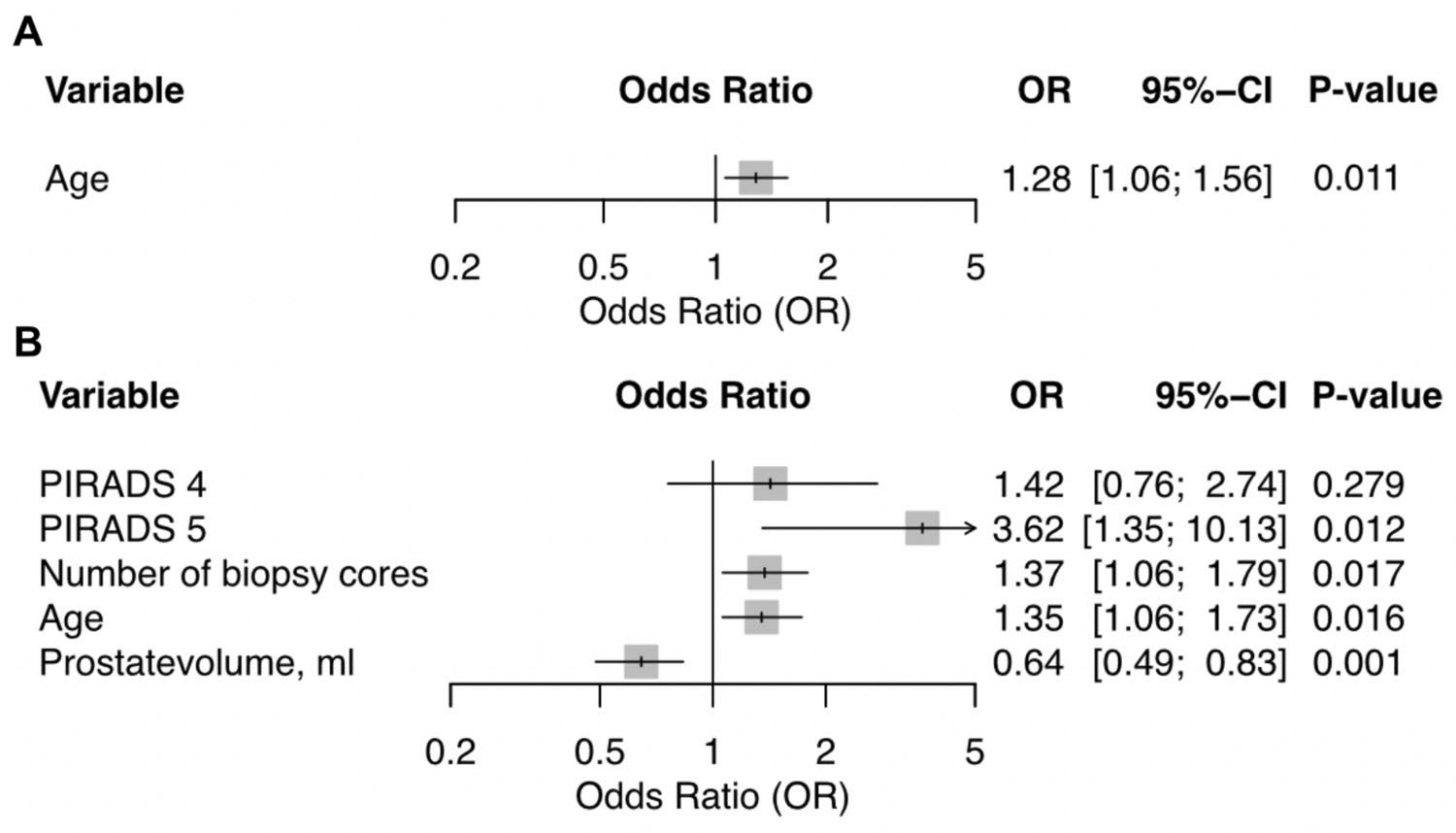
Positive biopsies are associated with clinical and radiological characteristics. Associations between **a** age (*n* = 496), and **b** PIRADS, number of biopsy cores, age, and prostate volume in milliliters (*n* = 298) with prostate biopsies positive for cancer using logistic regression analyses. PI-RADS 3 was used as reference. *PI-RADS* prostate imaging reporting and data system

To investigate predictors of positive biopsies, we performed logistic regression analysis (Fig. 4a, b). Older age, higher PI-RADS score, greater number of biopsy cores, and smaller prostate volume were all significantly associated with biopsy positivity. PI-RADS 5 lesions were associated with markedly increased odds of cancer detection compared to PI-RADS 3 (OR 3.6, 95% CI 1.35–10.13). Age and number of biopsy cores were also positively associated with cancer detection (*p* < 0.05), while increased prostate volume was inversely associated.

### Predictors of clinically significant cancer

**Fig. 5:**
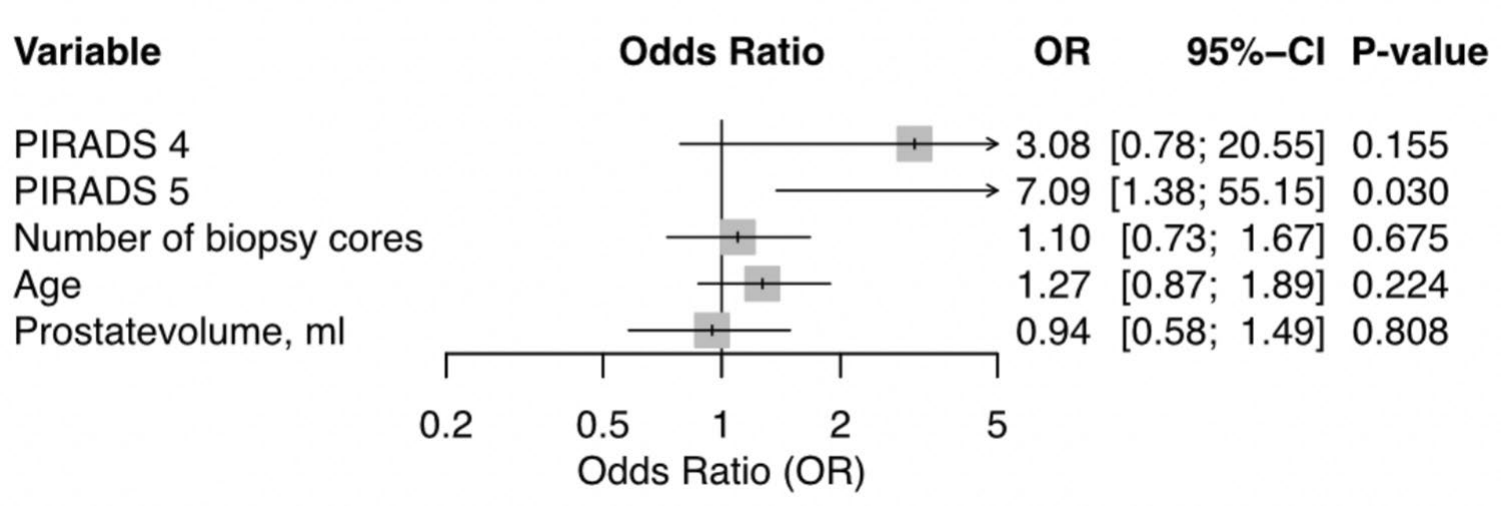
Significant prostate cancer biopsies are associated with radiological characteristics. Associations between **a** PI-RADS, number of biopsy cores, age, and prostate volume in milliliters with prostate biopsies positive for clinically significant prostate cancer (ISUP ≥ 3) using logistic regression analyses. Patients with positive biopsies (*n* = 121) were included. PI-RADS 3 was used as reference. *PI-RADS* prostate imaging reporting and data system, *ISUP* international society of urological pathology

To investigate predictors of clinically significant prostate cancer (ISUP ≥ 3), we analyzed data from patients with biopsy-confirmed cancer (Fig. 5). PI-RADS 5 lesions were significantly associated with csPCa compared to PI-RADS 3 (OR 7.1, 95% CI 1.38–55.15). In contrast, prostate volume, age, and number of biopsy cores were not significantly associated with csPCa in this subgroup (*p* > 0.05 for all). These results underscore PI-RADS as the strongest radiological predictor of clinically relevant disease.

### Tumor localization by prostatic region

To investigate the anatomical distribution of biopsy-detected tumors, we investigated the absolute numbers and percentages of detected tumors by prostatic region (Table 2). Cancer was most frequently detected in the basal left (*n* = 77) and least frequently in the basal right (*n* = 70) prostatic gland. Concordantly, the proportion of clinically significant cancers was highest in the basal left (34%)and lowest in the basal right (24%).

**Table 2:** Biopsy results according to the prostatic region

location	total cancers	clinically significant cancers	non-significant cancer	proportion significant
apical left	66	19	47	0.288
apical right	69	19	50	0.275
basal left	77	26	51	0.338
basal right	70	17	53	0.243

### Concordance with radical prostatectomy

**Fig. 6:**
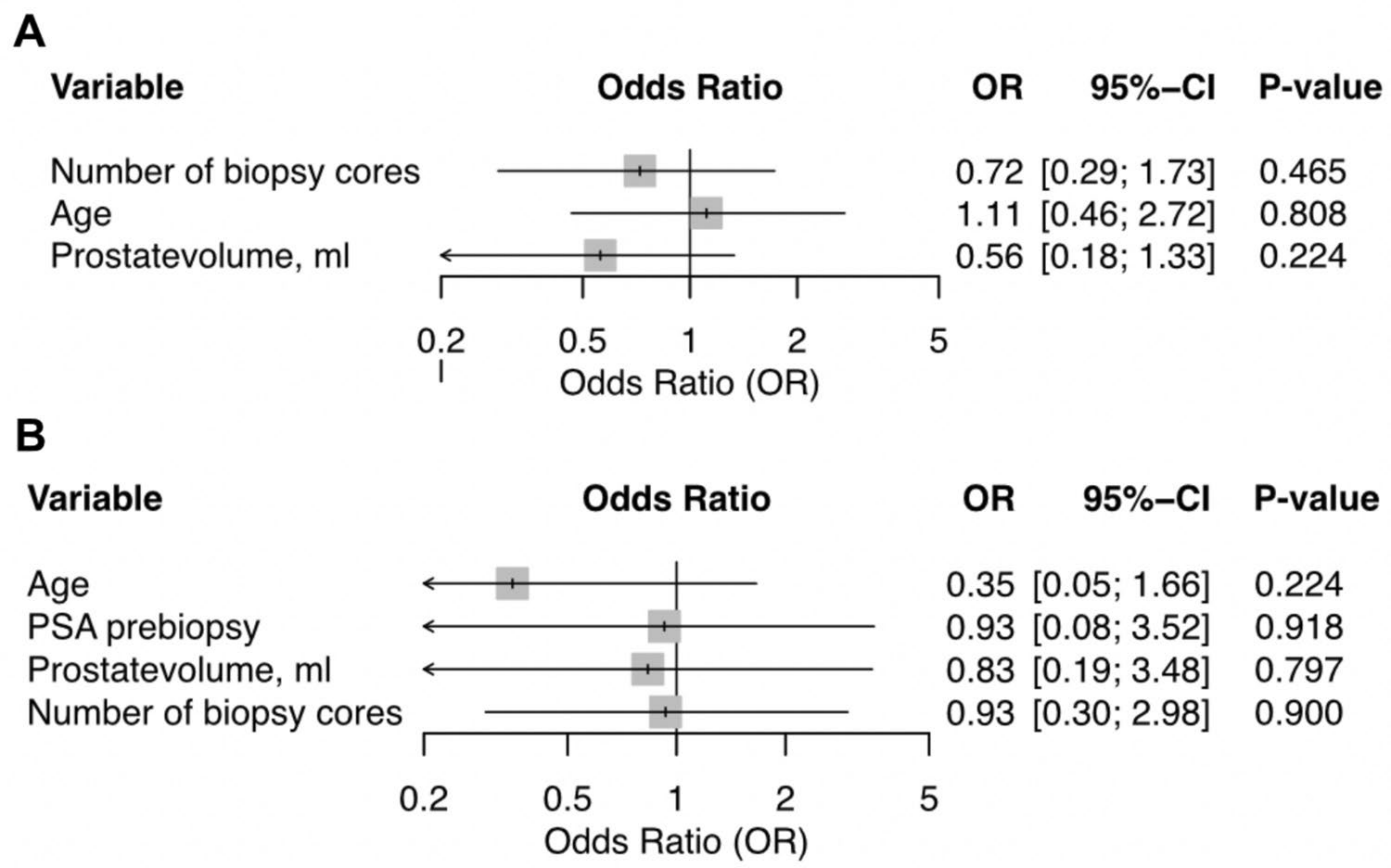
Association of clinical characteristics with upgrading at radical prostatectomy. Associations between **a** number of biopsy cores, age, and prostate volume in milliliters, **b** and total prebiopsy PSA with upgrading across any ISUP category. Patients who had a radical prostatectomy in-center were included leading to a total of **a** 26 or **b** 18 patients due to missingness of covariates. *PI-RADS* prostate imaging reporting and data system, *ISUP* international society of urological pathology

To assess the concordance of MRI-guided biopsy with final pathology, we analyzed 29 patients who underwent in-house radical prostatectomy (Table 3, 4). Most cancers were confirmed as ISUP 2 (52%) or ISUP 5 (21%) at prostatectomy (Table 1). Only one patient (3.6%) was upgraded from non-significant (ISUP 1–2) to significant disease (ISUP ≥ 3), suggesting high reliability in sampling csPCa. However, upgrading across any ISUP category occurred in 63% (17/27 patients with complete data). To investigate potential predictors of ISUP upgrading at prostatectomy, we performed logistic regression analyses (Fig. 6a, b). Neither PI-RADS score, age, PSA, number of biopsy cores, nor prostate volume were significantly associated with upgrading risk. This suggests that upgrading may reflect intrinsic histopathologic variability rather than clinical or imaging parameters.

**Table 3:** Baseline Characteristics and Biopsy Results**.**

	Overall
n	29
Age at biopsy (median (IQR, range))	67.4 (63–72, 55–77)
Prostatevolume, ml (mean (SD))	39.5 (17.2)
PSA, total, prebiopsy, ng/ml (median (IQR, range)	7.2 (5–10, 0,35–88,7)
Time between biopsy and radical prostatectomy, months (mean (SD))	19.2 (35.6)
positive biopsy (%)	17 (58.6)
significant cancer (%)	9 (31)
PI-RADS (%)	
3	3 (20)
4	11 (73.3)
5	1 (6.7)
ISUP, biopsy (%)	
1	6 (20.7))
2	2 (6.9)
3	4 (13.8)
4	3 (10.3)
5	2 (6.9)
ISUP, radical prostatectomy (%)	
1	3 (10.3)
2	15 (51.7)
3	3 (10.3)
4	2 (6.9)
5	6 (20.7)
any upgrading (%)	17 (63.0)

This table summarizes the demographic and clinical baseline characteristics of the study population. It includes patient age, prostate volume, and laboratory parameters. Additionally, imaging findings according to the PI-RADS system and the histopathological results of the prostate core biopsy are presented. The distribution of biopsy results across the different ISUP grades is clearly outlined

**Table 4:** Radical Prostatectomy Pathology.

	Overall
n	29
ISUP, radical prostatectomy (%)	
1	3 (10.3)
2	15 (51.7)
3	3 (10.3)
4	2 (6.9)
5	6 (20.7)
T-Stadium (%)	
pT2	9 (31)
pT3a	9 (31)
pT3b	9 (31)
pT4	1 (3.4)
Lymph node metastasis (%)	3 (10.3)
Lymphatic invasion (%)	2 (6.9)
Vascular invasion (%)	0 (0)
Perineural infiltration (%)	19 (73.1)
R0-Resection (%)	
R0	10 (34.5)
R1	19 (65.5)

This table presents the results of the histopathological analysis of the prostatectomy specimen. It reports the final ISUP grade, the pathological T stage according to the TNM classification, the surgical margin status, as well as information on lymph node involvement and vascular invasion

### Safety outcomes

Post-biopsy CT imaging was reviewed to assess procedural safety. Among the 496 patients, the overall complication rate was low. Localized minor bleeding was observed in 22 cases (4.4%), and in all instances, there was no evidence of hemorrhage requiring intervention. No major adverse events were recorded, confirming the favorable safety profile of MRI-guided biopsy in this cohort.

## Discussion

MRI-guided prostate biopsy demonstrates robust detection of clinically significant prostate cancer, consistent with previous reports across different targeting techniques [35–38]. PI-RADS 5 lesions consistently predict the highest likelihood of clinically significant tumors, reinforcing their value as a pre-biopsy stratification tool [5, 39]. Variables such as patient age, prostate volume, and number of biopsy cores appear to influence overall biopsy positivity but show limited utility in predicting tumor aggressiveness, a finding aligned with multicenter observations [31, 40].

The anatomical distribution of significant cancers, particularly in the basal and peripheral zones, aligns with prior literature, suggesting both biological tumor predilection and the influence of biopsy access routes on lesion detection [41]. Low rates of upgrading to clinically significant disease after radical prostatectomy in our cohort suggest a high concordance between MRI-guided biopsy and final pathology, consistent with other studies reporting minimal clinical impact of histopathologic upgrading despite frequent changes in ISUP grade [9, 42–45]. Nevertheless, upgrading across any grade remains common, emphasizing ongoing challenges in precise risk stratification, particularly for low- and intermediate-risk lesions, likely driven by tumoral heterogeneity rather than sampling error.

While the overall performance of MRI-guided biopsies is comparable to fusion- and cognitive-targeted approaches, critical evaluation of individual studies highlights potential limitations. For instance, Wegelin et al. reported promising detection rates, yet the study’s design introduces uncertainty regarding its generalizability. Operator experience in the MRI-targeted biopsy group was limited, with trainees performing procedures for only six months, whereas FUS- and cognitive-targeted biopsies were conducted by experienced urologists. Differences in median biopsy cores and variable detection of PI-RADS 4 lesions further complicate direct comparison. Additionally, the rate of false-negative cases was not clearly reported, restricting the ability to assess diagnostic sensitivity. These factors underscore the importance of cautious interpretation when comparing targeting techniques across heterogeneous study settings.

Safety outcomes in our cohort were favorable, with complication rates aligning with prior reports, confirming the low-risk profile of MRI-guided prostate biopsy [25, 42]. Taken together, our findings support the clinical reliability of MRI-guided in-bore biopsy for detecting high-grade prostate cancer while highlighting the influence of lesion characteristics, operator experience, and procedural variables on diagnostic yield. Future studies should continue to evaluate the relative merits of different targeting techniques in real-world settings, with attention to standardization and training to optimize detection while minimizing unnecessary sampling.

This study has several limitations. First, its retrospective, single-centre design limits generalisability. Second, PI-RADS versions 2.0 and 2.1 were not available for early cases, and changes in reporting standards over time may have introduced variability. Third, the relatively small subset of patients who underwent prostatectomy limits the statistical power of concordance analyses. Finally, potential interobserver variability in PI-RADS assessment and histopathological grading was not systematically addressed.

In conclusion, MRI-guided prostate biopsy demonstrated strong diagnostic performance and a favourable safety profile in a real-world setting. PI-RADS 5 was identified as the most robust predictor of clinically significant prostate cancer, whereas age, prostate volume, and the number of biopsy cores were associated with overall biopsy positivity without discriminating tumour aggressiveness. Compared with cognitive and MRI/US fusion-guided biopsy, the in-bore approach offers the specific advantage of real-time MRI visualization of both the lesion and the biopsy needle, enabling precise sampling of small, anterior, or difficult-to-target tumors while requiring fewer biopsy cores. This level of targeting accuracy may contribute to the high concordance observed between biopsy and final surgical pathology in our cohort. The low rate of clinically significant upgrading and the favorable safety profile support the routine integration of MRI in-bore biopsy into contemporary diagnostic pathways for prostate cancer. These findings reinforce the value of targeted biopsy strategies in enabling personalized treatment decisions and reducing the risk of overtreatment in men with suspected prostate cancer.

## Data Availability

No datasets were generated or analysed during the current study.

## Abbreviations

ADC: Apparent diffusion coefficient
CI: Confidence interval
csPCa: Clinically significant prostate cancer
CT: Computed tomography
DCE: Dynamic contrast-enhanced
DWI: Diffusion-weighted imaging
EAU: European association of urology
ESUR: European society of urogenital radiology
ISUP: International society of urological pathology
IQR: Interquartile range
mpMRI: Multiparametric MRI
MRI: Magnetic resonance imaging
N: Absolute number
OR: Odds ratio
PI-RADS: Prostate imaging reporting and data system
PSA: Prostate specific antigen
RPE: Radical prostatectomy
SD: Standard deviation
T1w: T1-weighted
T2w: T2-weighted
